# The Long Non-Coding RNA *SNHG12* as a Mediator of Carboplatin Resistance in Ovarian Cancer via Epigenetic Mechanisms

**DOI:** 10.3390/cancers14071664

**Published:** 2022-03-25

**Authors:** Cecilie Abildgaard, Luisa Matos do Canto, Cláudia Aparecida Rainho, Fabio Albuquerque Marchi, Naiade Calanca, Marianne Waldstrøm, Karina Dahl Steffensen, Silvia Regina Rogatto

**Affiliations:** 1 Department of Clinical Genetics, Lillebaelt University Hospital of Southern Denmark, 7100 Vejle, Denmark; cecilieabild@gmail.com (C.A.); luisa.matos.do.canto.alvim@rsyd.dk (L.M.d.C.); naiade.calanca@unesp.br (N.C.); 2 Department of Oncology, Lillebaelt University Hospital of Southern Denmark, 7100 Vejle, Denmark; karina.dahl.steffensen@rsyd.dk; 3 Department of Chemical and Biological Sciences, Institute of Biosciences, São Paulo State University (UNESP), Botucatu 18618-689, Brazil; claudia.rainho@unesp.br; 4 International Research Center, CIPE—C. Camargo Cancer Center, São Paulo 04002-010, Brazil; biomarchi@gmail.com; 5 Department of Pathology, Lillebaelt University Hospital of Southern Denmark, 7100 Vejle, Denmark; marianne.waldstroem@rsyd.dk; 6 Institute of Regional Health Research, Faculty of Health Sciences, University of Southern Denmark, 5000 Odense, Denmark

**Keywords:** ovarian cancer, lncRNA, drug resistance, chemotherapy, DNA methylation, transcriptomic analysis

## Abstract

**Simple Summary:**

Epithelial ovarian cancer is a lethal malignancy in which recurrence and therapy resistance are the major causes of death. We investigated the transcriptome and DNA methylation profile of ovarian cancer cell lines sensitive and resistant to carboplatin, aiming to identify genes associated with therapy resistance. We focused on long non-coding RNAs (lncRNAs), known as epigenetic regulators of several cellular and biological processes. We found 11 lncRNAs associated with carboplatin resistance, including *SNHG12* (small nucleolar RNA host gene 12), also confirmed in an external dataset (The Cancer Genome Atlas). *SNHG12* gene silencing increased the sensitivity to carboplatin, giving evidence that this lncRNA contributes to resistance to carboplatin in ovarian cancer cell lines. We also demonstrated that *SNHG12* could control the expression of nearby genes probably by altering epigenetic markers and modifying the transcript levels.

**Abstract:**

Genetic and epigenetic changes contribute to intratumor heterogeneity and chemotherapy resistance in several tumor types. LncRNAs have been implicated, directly or indirectly, in the epigenetic regulation of gene expression. We investigated lncRNAs that potentially mediate carboplatin-resistance of cell subpopulations, influencing the progression of ovarian cancer (OC). Four carboplatin-sensitive OC cell lines (IGROV1, OVCAR3, OVCAR4, and OVCAR5), their derivative resistant cells, and two inherently carboplatin-resistant cell lines (OVCAR8 and Ovc316) were subjected to RNA sequencing and global DNA methylation analysis. Integrative and cross-validation analyses were performed using external (The Cancer Genome Atlas, TCGA dataset, *n* = 111 OC samples) and internal datasets (*n* = 39 OC samples) to identify lncRNA candidates. A total of 4255 differentially expressed genes (DEGs) and 14529 differentially methylated CpG positions (DMPs) were identified comparing sensitive and resistant OC cell lines. The comparison of DEGs between OC cell lines and TCGA-OC dataset revealed 570 genes, including 50 lncRNAs, associated with carboplatin resistance. Eleven lncRNAs showed DMPs, including the *SNHG12*. Knockdown of *SNHG12* in Ovc316 and OVCAR8 cells increased their sensitivity to carboplatin. The results suggest that the lncRNA *SNHG12* contributes to carboplatin resistance in OC and is a potential therapeutic target. We demonstrated that *SNHG12* is functionally related to epigenetic mechanisms.

## 1. Introduction

Ovarian cancer (OC) is one of the most lethal malignancies in women and accounts for more than 200,000 deaths worldwide annually [1]. OC is histologically divided into at least five subtypes showing distinct etiological, morphological, clinical, genetic, and epigenetic aspects [2,3]. Epithelial ovarian cancer (EOC) accounts for ~90% of cases and includes serous, endometrioid, clear cell, and mucinous carcinomas. High-grade serous OC is the most commonly diagnosed, and in more than 70% of cases presents advanced stages (International Federation of Gynecology and Obstetrics, FIGO, stage III or IV) [2,4,5].

Primary care of OC patients is based on surgery and adjuvant chemotherapy. Despite the variety of OC subtypes, pointing in the direction that each subtype should potentially be treated differently, OC is treated as a single disease. Standard first-line treatment includes primary cytoreductive surgery followed by adjuvant chemotherapy with platinum-based drugs, namely carboplatin or cisplatin, and a taxane, such as paclitaxel or docetaxel [6,7]. Initially, more than 80% of the women benefited from the first-line treatment. However, within two years from diagnosis, most patients with advanced disease experience recurrence due to treatment resistance [7]. Ovarian cancer that recurs within six months of completion of initial therapy is considered “platinum-resistant”, while OC that recurs after six months of completion of initial therapy is considered “platinum-sensitive” [8]. In advanced stages of the disease, the efficacy of different therapies decreases with each recurrence, and the patients face an abysmal prognosis of less than 25% 5-year survival [9,10,11]. Cancer recurrence and therapy resistance are considered the major causes of death. Therefore, it is critically urgent to understand the pathobiology of this aggressive disease better and provide more precise and effective treatments.

Among the drugs used in recurrent OC, PARP (poly- (adenosine diphosphate-ribose) polymerase) inhibitors have demonstrated prognostic impact in patients with pathogenic/likely pathogenic *BRCA1/BRCA2* variants or genomic instability [12,13]. After primary chemotherapy, PARP inhibitors have also been used as maintenance therapy [12]. The anti-angiogenic monoclonal antibody bevacizumab, which binds to vascular endothelial growth factor, has been approved to treat EOC, but no significant difference in overall survival and limited improvement in progression-free survival has been described [14]. Immunological therapies, anti-angiogenic agents, combination therapies, and treatment based on genetic alterations (targeted therapies) have been intensively studied to improve the survival and quality of life of OC patients.

The heterogeneity of the disease and the molecular complexity of the genetic and epigenetic alterations promoted during tumor progression or due to the selective pressure caused by the treatment is rarely captured and taken into consideration in the clinical setting [15]. The molecular alterations leading to platinum resistance rarely involve single nucleotide mutations in known drivers or resistance-associated genes. Instead, the resistance appears to arise from a number of highly patient-specific alterations such as aberrant DNA methylation, gene amplifications, reversion of *BRCA1/2* (BRCA1 and BRCA2 DNA repair associated) mutations, chromosomal rearrangements, and differential expression of non-coding RNAs (ncRNAs) [10,16,17,18].

The ncRNAs, which are involved in transcriptional and post-transcriptional regulation, constitute an additional level of complexity in cancer drug resistance. Among them, long non-coding RNAs (lncRNA) have been drawing attention for their function as regulators of transcription and chromatin remodeling. LncRNAs include transcripts longer than 200 nucleotides that form complex structures that interact with DNA, RNA, or proteins to modulate gene expression and cellular signaling [19]. LncRNAs are expressed in lower levels compared to mRNA, control the expression pattern during the development and cell differentiation, and are often cell-type and cancer-type specific [20]. For instance, *SNHG10* (small nucleolar RNA host gene 10) overexpression significantly suppressed the tumorigenesis and epithelial-mesenchymal transition (EMT) of EOC [21]. *SNHG10*/miR-200a-3p/*BIN1* axis was reported as a predictive biomarker and therapeutic target in OC [21]. The lncRNA *HOTAIR* has been described as altered in different tumor types. In EOC, overexpression of *HOTAIR* was associated with poor prognosis and pro-metastatic effects, which are at least partially exerted by regulating the expression of metalloproteinases and EMT-related genes [22]. Recent reviews have summarized the role of lncRNAs in tumor progression and invasion [17,23].

High-throughput transcriptome studies have identified differential expression patterns of lncRNAs associated with disease recurrence and platinum resistance [24,25,26]. Furthermore, several aberrantly expressed lncRNAs in resistant OC cells have been functionally related to resistance mechanisms, including increased cell division, improved DNA repair, upregulation of drug transporters, or reduced susceptibility to apoptotic stimuli [17,27,28,29].

A well-known mechanism of transcriptional regulation is DNA methylation. The direct interaction between lncRNAs and proteins of the epigenetic machinery, such as chromatin modifiers and DNA methyltransferases, has emerged as an additional link between lncRNAs and tumorigenesis. Thus, knowledge of the interplay between lncRNAs with epigenetic modification and transcriptional regulation significantly contributes to understanding the mechanisms involved in gene regulation and unravels robust markers associated with resistance to therapy. Importantly, lncRNA targeting is one of the only therapeutic approaches that can upregulate tumor suppressor genes endogenously in a locus-specific manner [30]. Despite these promising findings and the large number of lncRNAs in the human genome, only a few of them have been well characterized, annotated, and explored. It is critical to elucidate the actual functions of cancer-related lncRNAs and their association with therapy resistance in OC. Moreover, the functional relationship between lncRNA expression and epigenetic regulation is not yet well-understood.

Several studies have demonstrated that lncRNAs are associated with tumor progression and chemotherapy resistance. However, the implications of DNA methylation changes, lncRNAs, and their association with chemotherapy response in OC are poorly explored. Herein, using RNA sequencing (RNA-seq) and DNA methylation analyses in OC cell lines, we identified the lncRNA *SNHG12* (small nucleolar RNA host gene 12) as a candidate biomarker of response to carboplatin in OC and investigated its functional mechanisms.

## 2. Materials and Methods

### 2.1. Cell Lines and Patient Samples

A panel of six OC cell lines (IGROV1, OVCAR3, OVCAR4, OVCAR5, OVCAR8, and Ovc316) were included in this study. The cell lines OVCAR3, OVCAR5, and Ovc316 were kindly provided by Robert Strauss, Danish Cancer Society, Denmark, and the cell lines IGROV1, OVCAR4, and OVCAR8 were donated by Wan Lam, University of British Columbia, Vancouver, Canada. The cell lines were cultured in RPMI medium (Sigma Aldrich, Saint Louis, MO, USA) (cell lines IGROV1, OVCAR3, and OVCAR4) or Dulbecco’s modified Eagle medium (DMEM, Sigma Aldrich) (OVCAR5, OVCAR8, and Ovc316), as recommended. The media were supplemented with 10% fetal bovine serum (FBS) (Biological Industries, Beit HaEmek, Israel), and 1% antibiotic-antimycotic (10,000 IU/mL penicillin, 10 mg/mL streptomycin, and 25 µg/mL of Amphotericin B—Thermo Fisher, Waltham, MA, USA). After confluence, cells were treated with 0.05% trypsin/0.02% EDTA (Sigma Aldrich) and replicated. We tested cell cultures for mycoplasma contamination using a PCR-based assay once per month. The mutation profile of cancer-related genes described in these cell lines was confirmed by Sanger sequencing (Table S1).

High-grade serous ovarian tumor tissue samples (*n* = 39, FIGO stages III–IV) were obtained from patients who underwent surgical resection (mean age 69.46 ± 9.30, ranging from 46 to 83 years old) at Horsens Hospital, DK, between 2005 and 2007. Follow-up and progression-free survival (PFS) data were retrieved from the Danish Health Registries and research databases of the Department of Oncology, University Hospital of Southern Denmark, Vejle, DK. The samples were classified according to the World Health Organization (WHO) classification of female genital tumors [31]. Recurrence within 12 months after the last platinum treatment was defined as platinum resistance. All patients were included at the time of primary diagnosis and followed after first-line treatment. PFS was defined as the interval between the date of surgery and the date of disease progression or death by any cause. Overall survival (OS) was defined as the interval between the date of the treatment and death by any cause. In addition to tumor samples, eight normal ovarian tissues were obtained from individuals submitted to surgery for other causes than cancer and used as controls in the RT-qPCR experiments. The Table S2 summarizes the clinical data of the patients included in our study.

The study was conducted in accordance with the Declaration of Helsinki ethical guidelines and approved by the National Ethics Committee (case no. #1803521) and the Danish Data Protection Agency (19/48213). Informed consent was obtained from all patients prior to sample collection.

### 2.2. Establishment of Resistant Cell Lines

Carboplatin-sensitive cells (IGROV1, OVCAR3, OVCAR4, and OVCAR5) (Table S1) were exposed to increasing concentrations of carboplatin following an intermittent schedule with phases of recovery between the treatments. The baseline schedule consisted of 4 days of treatment with 1 µM of carboplatin in the culture media followed by 3 recovery days in media without carboplatin. For cell lines tolerating this treatment well, the concentration was increased gradually (1, −5, −10, or −15 µM) when the cells reached high confluency. For cell lines not tolerating the treatment well, the recovery was extended, and the low dose was maintained. The exact treatment regimen was dependent on the growth characteristics and drug response of the individual cell lines. In general, resistance was developed following 2–4 months of treatment. Acquired resistance was confirmed by IC_50_ measurements before and after this period using a crystal violet assay for determining the viability of cultured cells as previously described [32,33].

### 2.3. RNA Extraction and Sequencing

Total RNA was isolated with the RNeasy mini kit (Qiagen, Valencia, CA, USA) according to the manufacturer’s recommendations. The RNA purity and quantity were measured with a nanodrop spectrophotometer (Thermo Fisher Scientific, Waltham, MA, USA), and the integrity was assessed with RNA screen tape on a 2200 TapeStation (Agilent, Santa Clara, CA, USA).

Libraries were generated using 500 ng of total RNA and prepared using the TruSeq Stranded Total RNA Library Prep Gold kit (Illumina, San Diego, CA, USA), following the manufacturer’s protocol. Integrated DNA Technology (IDT) for TruSeq RNA UD Indexes was added for multiplexing, and samples were paired-end sequenced with 2 × 100 bp on the NovaSeq 6000 system (Illumina) using the S1 Reagent Kit v1.5 (200 cycles), as recommended by the supplier. RNA-seq data quality control was performed using FastQC and MultiQC [34,35]. Reads were aligned using STAR (v.2.7.6a) [36], and the gene read count was performed with HTSeq-count using the Ensembl human genome assembly GRCh38, release 99. The final count matrix consisted of 60,676 genes (raw counts), including those with no counts. The differential expression analysis between carboplatin-sensitive and carboplatin-resistant cell lines was performed in the Galaxy bioinformatics platform using the default settings of the EdgeR package (including normalization TMM) [37,38]. The transcriptome data are deposited in the Gene Expression Omnibus (GEO) database (GSE198077).

### 2.4. Pathway Enrichment Analysis

We performed enrichment analysis using our list of DEGs to identify overrepresented pathways unique to sensitive and resistant OC cell lines. The analysis was performed using the Kyoto Encyclopedia of Genes and Genomes 2021 Human database via the Enrichr analysis tool [39].

### 2.5. DNA Extraction and DNA Methylation Profiling

Genomic DNA was extracted using DNeasy Blood and Tissue Kit (Qiagen, Valencia, CA, USA). A total of 210 ng of DNA was sodium bisulfite converted with the EZ DNA Methylation-Gold™ Kit (Zymo Research, Irvine, CA, USA), according to the manufacturer’s recommendations. The genome-wide methylation status of each sample was assessed using the Infinium MethylationEPIC 850 K BeadChip (Illumina, USA). The DNA methylation data is available at the GEO database (GSE198077). The microarrays were scanned using the Illumina HiScan system, and the data were processed using R language and analyzed as previously described [40]. For methylation data analysis, fluorescence intensity data (.IDAT) files were analyzed using the minfi R package [41]. Quality control, the *p*-value for all probes, background noise detection, and adjustment were performed using the same package. Differences Type-I and Type-II probes adjustment and normalization of β values were performed using the Beta Mixture Quantile dilation (BMIQ) model [42]. Batch effects were removed using the package SVA [43]. Probes with low quality (detection *p*-value > 0.05), mapped in X/Y chromosomes, and cross-reactive were filtered out, and those mapped to SNPs were removed (package minfi) [41,44]. Differentially methylated CpG positions (DMPs) were identified using the R package limma [45]. Probes with *p*-value < 0.01 and |∆β| > 0.1 were considered significant and annotated using the Illumina manifest file.

### 2.6. RNA-seq and DNA Methylation Integrative Analysis

Only lncRNAs showing differential expression and DNA methylation were selected for further analysis. Pearson correlation test (r values with *p* < 0.05) was applied to identify probes and genes/lncRNAs with positive (r+) or negative (r-) correlation between DNA methylation and gene expression.

### 2.7. External Data from The Cancer Genome Atlas

External RNA-seq data from The Cancer Genome Atlas (TCGA) was used as a validation dataset. A total of 111 RNA-seq gene count files from patients with high-grade serous ovarian cancer (HGSC) were downloaded from the Genomic Data Commons Data Portal (TCGA-OV project). Only patients who received treatment with carboplatin were selected from this data set. The data files were created using a similar bioinformatics pipeline to the one applied in the analysis of our experimental data (STAR-HTseq count). Clinical information was collected from a published study [46]. Based on PFS, the patients were categorized into either sensitive (PFS > 12 months, *n* = 69) or resistant (PFS < 12 months, *n* = 42) to carboplatin. Data normalization and differential expression analysis between sensitive and resistant samples were performed in the Galaxy bioinformatics platform using EdgeR [37,38].

### 2.8. Criteria Used to Select lncRNA for Functional Assays

We selected lncRNAs overexpressed in resistant compared to sensitive cell lines. We filtered out lncRNAs not associated with differential DNA methylation and lncRNAs that were downregulated in the resistant OC samples from the TCGA cohort. Among the remaining lncRNAs with the highest expression, we investigated their roles in cancer and response to therapy available in the literature. The final candidate, *SNHG12*, was further investigated by determining its expression levels in the internal cohort of 39 HGSC samples. Next, siRNA experiments were performed in selected OC cell lines. Bioinformatics analyses were also carried out using online lncRNA databases for investigating *SNHG12* subcellular localization (LncACT.db 3.0 available at http://bio-bigdata.hrbmu.edu.cn/LncACTdb, last accessed on 10 January 2022), regulatory relationships (LncRNADisease 2.0 available at http://www.rnanut.net/lncrnadisease, last accessed on 10 January, 2022), and co-expressed genes (Lnc2Cancer 3.0 available at http://bio-bigdata.hrbmu.edu.cn/lnc2cancer/, last accessed on 10 January 2022) [47,48,49].

### 2.9. RNA Extraction and RT-qPCR Assay

Total RNA was isolated from frozen ovarian tissues (39 tumor and 8 non-tumor samples) and OC cell lines using the RNeasy mini kit (Qiagen, Hilden, Germany). Reverse transcription of 1 µg RNA was carried out with the High-Capacity RNA to cDNA kit (Thermo Fisher, Waltham, MA, USA) according to the manufacturer’s instructions. Quantitative PCR was performed using TaqMan Fast Advanced Master Mix and TaqMan probes (assay ID: Hs00939627 for reference gene *GUSB* and assay ID: Hs00414754 for the target gene *SNHG12*). The RT-qPCR amplifications were performed in a QuantStudio 12K Flex Real-Time PCR System (Thermo Fisher, Waltham, MA, USA) with the following parameters: 50 °C for 2 min, 95 °C for 2 min, 40 cycles at 95 °C for 1 s, and 60 °C for 20 s. The relative expression in each sample was calculated using the 2^−ΔΔCt^ method [50].

### 2.10. Knockdown of lncRNA SNHG12 and Carboplatin Exposure

Four OC cell lines (IGROV1 and its carboplatin-resistant counterpart IGROV1-R1, OVCAR8, and Ovc316) were selected for knockdown experiments of the lncRNA *SNHG12* using a small interference RNA (siRNA) approach. Cells were seeded into flat-bottom 6-well (3 × 10^5^ cells/well) and 96-well (1 × 10^4^ cells/well) tissue culture plates and maintained at 37 °C under 5% CO_2_. Transfections were carried out with ~60% confluency in each of the four cell lines using Opti-MEM I Reduced Serum Medium, GlutaMAX Supplement, and Lipofectamine RNAiMAX Transfection Reagent (Thermo Fisher, Waltham, MA, USA), according to the manufacturer’s recommendations. The Silencer Select Negative Control siRNA #1 (cat# 4390843) and *SNHG12* siRNA (cat#4392422, ID: n543460) were used at 1 pmol (96-well plate) and 25 pmol (6-well plate) per well, respectively (Thermo Fisher, Waltham, MA, USA). Transfection efficiency was determined using BLOCK-iT Alexa Fluor Red Fluorescent Oligo (Thermo Fisher, Waltham, MA, USA). After 48 h incubation, the medium was replaced with the growth medium only or medium containing two different concentrations of carboplatin (10 µM or 50 µM). Triplicates of each condition were analyzed in two independent experiments. The step-by-step workflow used to evaluate the *SNHG12* silencing is shown in Figure S1.

### 2.11. Cell Viability Assays

Following carboplatin treatment, cell viability was measured using the standard protocol of CellTiter Blue Viability Assay (Promega, Madison, WI, USA) at 24 h, 72 h, and 96 h time points. Fluorescence was recorded at 530/590 nm using the Synergy HT plate reader (BioTek, Winooski, VT, USA). Reading corrections were performed using wells with no cells treated under the same conditions.

### 2.12. Data Processing and Statistical Analyses

Data were generated from at least two independent experiments with triplicates. Graphical and statistical analyses were performed using Graph Pad Prism 9.0 (Graph Pad Software, San Diego, CA, USA) and Galaxy bioinformatics platform [37]. Clusters were generated using Euclidean distance and complete linkage. Group data are reported as mean ± SD. Analysis of variance (ANOVA with Tukey’s multiple comparisons test and Fisher’s exact test) was used to compare cell viability among the different conditions (siRNA versus scrambled RNA) and time points (24 h, 72 h, and 96 h). Survival analyses were performed using Kaplan–Meier and log-rank methods. Statistical significance was considered with *p*-values ≤ 0.05.

## 3. Results

### 3.1. Carboplatin Response in Ovarian Cancer Cell Lines and Establishment of Carboplatin-Resistant Derived Subpopulations

Among the six OC cell lines, four were classified as carboplatin sensitive (IGROV1, OVCAR3, OVCAR4, and OVCAR5), and two (OVCAR8 and Ovc316) were inherently resistant (IC_50_ values above 32 µM). Carboplatin acquired resistance was observed in derived subpopulations from IGROV1 (IGROV1-R1), OVCAR3 (OVCAR3-R1 and OVCAR3-R2), and OVCAR5 (OVCAR5-R1). OVCAR4 presented high sensitivity to carboplatin and, therefore, this cell line was not used in the functional assays. The resistance to carboplatin was validated by an increase in the IC_50_ from 4–17 µM up to 28–44 µM (Table S1 and Figure S2A). Two resistant subpopulations were derived in parallel from OVCAR3 (OVCAR3-R1 and OVCAR3-R2) and exhibited a right shift of the dose-response curves of carboplatin (Figure S2B). OVCAR3-R1 and OVCAR3-R2 were established from independent cultures.

### 3.2. Differential Expression Profiles Associated with Carboplatin Resistance in Ovarian Cancer Cell Lines and Ovarian Cancer Tissues

**Figure 1:**
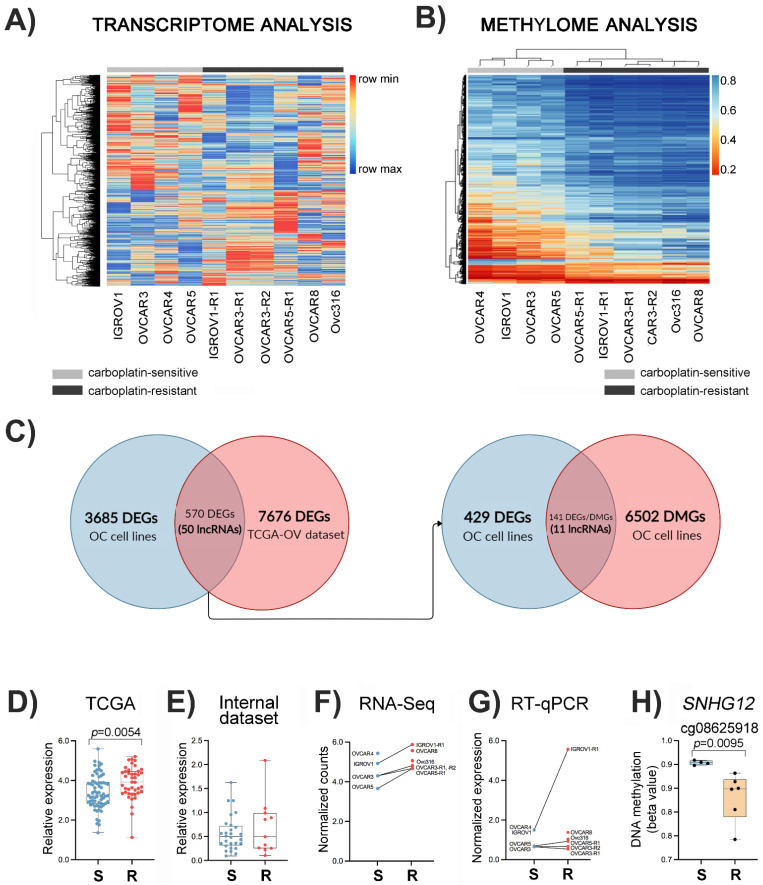
Identification of differentially expressed and methylated lncRNAs associated with carboplatin resistance in ovarian cancer (OC) cell lines. (**A**) Clustering analysis with normalized read counts of 4255 significantly differentially expressed genes (DEGs) in cell lines sensitive and resistant to carboplatin. (**B**) Heatmap with the 5721 DMPs obtained after comparing the resistant and sensitive cell lines. (**C**) The Venn diagram shows the intersection between DEGs detected in the ten OC cell lines and TCGA-OV dataset when comparing carboplatin-sensitive and -resistant specimens, including 50 lncRNAs. Integrative transcriptome and methylome analysis revealed that 11 lncRNAs were DEGs and differentially methylated genes (DMGs). The expression levels of the lncRNA *SNHG12* in OC tissues and cell lines, classified as sensitive or resistant to chemotherapy, are shown at the bottom. (**D**) The first graph presents data retrieved from TGCA-OV (*n* = 111 cases, 69 sensitive and 42 resistant), and (**E**) the second was obtained from an internal cohort analyzed by RT-qPCR (*n* = 39 cases, 28 sensitive and 11 resistant). The comparison of lncRNA *SNHG12* expression levels determined by RNA-Seq and RT-qPCR analysis in OC cell lines and their carboplatin-resistant counterparts are shown in (**F**,**G**), respectively. (**H**) DNA methylation of cg08625918 (mapped on *SNHG12* locus) in carboplatin-sensitive and -resistant OC cell lines. (S, carboplatin-sensitive and R, carboplatin-resistant OC cell lines.)

To assess differential gene expression between the sensitive and resistant OC cells, RNA sequencing was performed in the four cell lines with acquired in vitro resistance to carboplatin (IGROV1-R1, OVCAR3-R1, OVCAR3-R2, and OVCAR5-R1), four sensitive cell lines (IGROV1, OVCAR3, OVCAR4, and OVCAR5), and the two inherently carboplatin-resistant OC cell lines (Ovc316 and OVCAR8). Genes differentially expressed were determined by comparing the sensitive with the resistant cell lines. This comparison revealed 4255 differentially expressed genes (DEGs; *p* < 0.05), among which 1913 were upregulated and 2342 downregulated in carboplatin-resistant OC cells. The normalized read counts considering FDR < 0.05 and logFC < −1.5/> 1.5 revealed 86 differentially expressed genes comparing cell lines sensitive and resistant to carboplatin. Supervised clustering analysis of the significant genes between resistant and sensitive OC cells is depicted in Figure 1A. The two resistant subpopulations derived from OVCAR3, OVCAR3-R1 and R2, exhibited very similar transcriptional profiles.

The pathway enrichment analysis using the most significant genes with fold change >1.5 between sensitive and resistant cells revealed 25 pathways upregulated and 117 downregulated in resistant cases (*p* < 0.05). Interestingly, several of the downregulated genes were involved in epigenetic regulation of gene expression, including DNA methylation and histone post-translational modifications (Table S3).

To select the DEGs that were also representative of resistance to carboplatin in a cohort of 111 HGSC patients from TCGA, we used differential expression analysis of carboplatin-sensitive (*n* = 69) and -resistant (*n* = 42) patients, identifying 8246 DEGs (*p* < 0.05) (Figure 1C). The comparison of DEGs between OC cell lines and OC primary tissues from the TCGA dataset resulted in 570 genes altered in both cohorts (Figure 1C), with 304 showing the same direction. Among those, 110 upregulated and 194 downregulated genes were associated with carboplatin resistance. Among the 304 DEGs overlapped between resistant cell lines and resistant OC patients from the TCGA dataset, 50 transcripts were categorized as lncRNAs, with 34 upregulated and 16 downregulated transcripts.

### 3.3. Differential Methylation Profile Associated with Carboplatin Resistance in Ovarian Cancer Cell Lines

Global DNA methylation was determined with the Infinium MethylationEPIC 850K array (Illumina) for the ten cell lines subjected to RNA sequencing. The comparison of resistant and sensitive cell lines revealed 14,529 differentially methylated CpG positions (DMPs, *p* < 0.05), 13,023 hypermethylated and 1506 hypomethylated in resistant OC cells. Clustering of the most significant DMPs (5721 positions with *p* ≤ 0.01 and |∆β| ≥ 0.1) clearly showed that resistant and sensitive cells have a distinct methylation profile (Figure 1B).

### 3.4. Integrative Data Analysis

**Figure 2:**
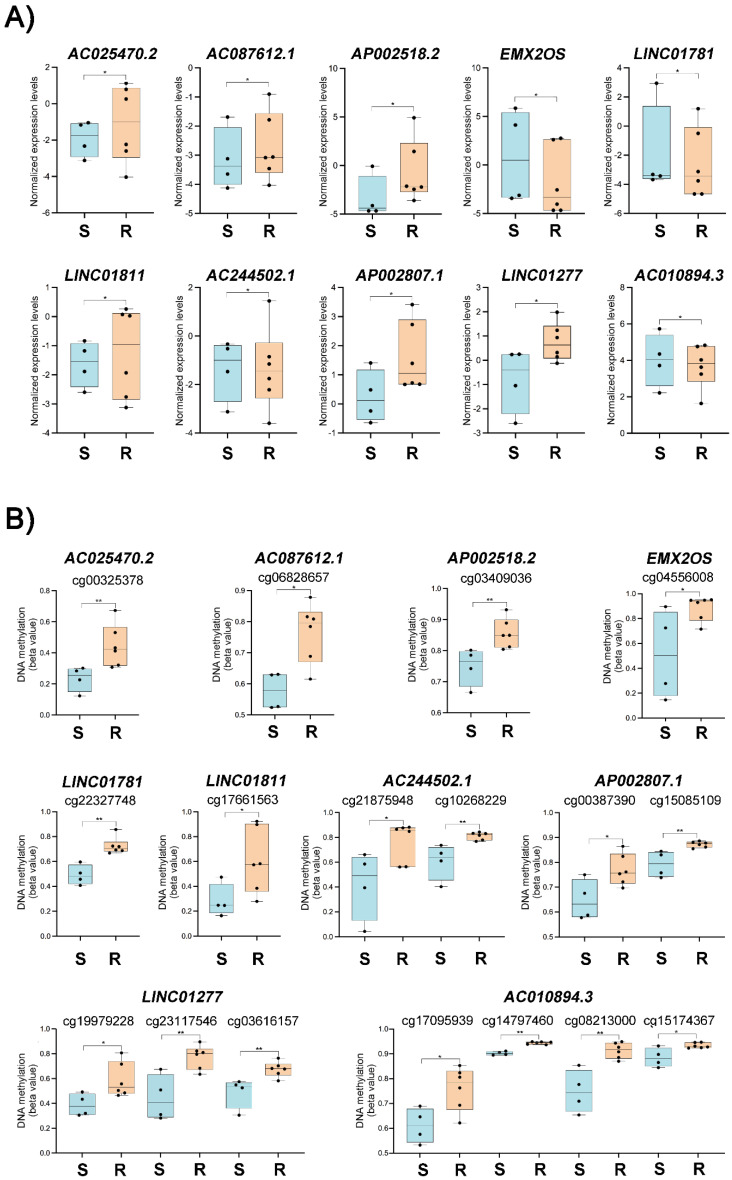
lncRNAs associated with carboplatin resistance in ovarian cancer (OC) cell lines. (**A**) Expression levels of ten out of eleven selected long non-coding RNAs were detected as differentially expressed and methylated in OC cell lines according to sensitivity or resistance to carboplatin. (**B**) Comparison of DNA methylation levels (beta value) of differentially methylated positions (CpG) located nearby to the ten selected long non-coding RNAs in sensitive and resistant cell lines. (*) *p* < 0.05 and (**) *p* < 0.01 (non-parametric *t* test). (S, carboplatin-sensitive and R, carboplatin-resistant OC cell lines).

The transcriptome and DNA methylation data integration unveiled 11 differentially expressed and methylated lncRNAs (Figure 1C) associated with 18 DMPs (Table S4), including the lncRNA *SNHG12*. The lncRNA *SNHG12* was selected for further analysis due to its increased expression in HGSC-TCGA patients (Figure 1D) and in resistant OC cell lines (Figure 1F,G). The cell lines OVCAR3-R1, OVCAR3-R2, IGROV1-R1, and OVCAR5-R1 showed increased *SNHG12* expression compared to their carboplatin-sensitive counterparts (Figure 1G). Additionally, this lncRNA was associated with a hypomethylated CpG in carboplatin-resistant cell lines (Figure 1H), showing a negative correlation with *SNHG12* expression levels. Figure 2A,B depict the difference in expression levels and beta values of the ten lncRNAs and DMPs, respectively, according to sensitivity to carboplatin in OC cell lines. Six of these lncRNAs were upregulated in resistant cells, as shown in Figure 2A. The downregulated lncRNAs, including *EMX2OS*, presented hypermethylated CpG positions (Figure 2B)

### 3.5. Platinum Resistance-Associated Genes

The list of 4255 DEGs and 14,529 DMPs detected in our panel of OC cell lines was compared with the platinum response-related gene list (937 genes/proteins) recently curated by Huang et al. [51]. We found 254 DEGs and 142 DMPs overlapping with the database entries (Table S5).

### 3.6. The lncRNA SNHG12 Is Overexpressed in Ovarian Cancer

The expression of the lncRNA *SNHG12* was also quantified in an independent cohort of 39 HGSC and 7 normal ovarian tissues (internal dataset). We found no significant differences between patients sensitive and resistant to carboplatin, possibly due to the limited number of resistant patients (Figure 1C). To evaluate the clinical significance of *SNHG12* expression in OC patients, we divided the cohort into *SNHG12* high expression and low expression groups according to the cutoff value, which was defined as quartiles. As demonstrated in Figure S3, patients from HGSC-TCGA with higher expression of *SNHG12* presented shorter PFS (*p* = 0.03). However, no significant difference was observed for OS in this cohort and for PFS (6 or 12 months) and OS in our internal dataset.

### 3.7. Functional Analysis of the lncRNA SNHG12

**Figure 3:**
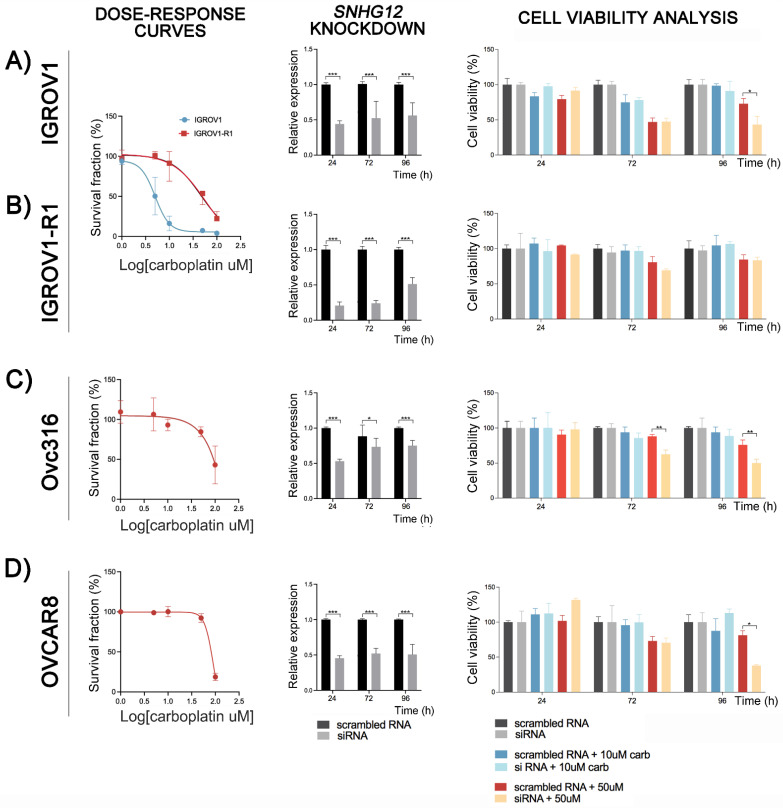
Effect of *SNGH12* knockdown on the cell viability of ovarian cancer (OC) cell lines exposed to carboplatin. Dose-response curves of carboplatin treatment, the efficiency of the siRNA targeting the lncRNA *SNHG12,* and its effects on cell viability after carboplatin treatment are shown for the IGROV1 and its induced resistant-counterpart IGROV1-R1 (**A**,**B**), and in intrinsically resistant Ovc316 (**C**) and OVCAR8 (**D**), respectively. The cells were exposed to scrambled RNA or *SNHG12* siRNA and treated with carboplatin (10 µM or 50 µM) during 24, 72 and 96 h. Cytotoxicity was determined by the CellTiter Blue Viability Assay. Knockdown led to a reduction of the relative expression levels of the lncRNA *SNHG12* compared with the scrambled RNA after 24, 72 and 96 h in the IGROV1/IGROV1-R1 (**A,B**), Ovc316 (**C**), and OVCAR8 (**D)** cells. (*) *p* < 0.05; (**) *p* < 0.001; (***) *p* < 0.0001.

We investigated the function of the lncRNA *SNHG12* on the carboplatin resistance in OC cells. The inherently resistant Ovc316 and OVCAR8 and the pair IGROV1 (sensitive) and IGROV1-R1 (IGROV1-derived carboplatin-resistant subpopulation) were transfected with si-*SNHG12*. Carboplatin dose-response curves were confirmed by cell viability assay before knockdown experiments (Figure 3A–D). The RT-qPCR results showed down-regulation of *SNHG12* in all cells transfected with siRNA-*SNHG12*. *SNHG12* inhibition increased the sensitivity to carboplatin of IGROV1, Ovc316, and OVCAR8 cells as indicated by reduced viability after 96 h of exposure to 50 μM carboplatin in the siRNA transfected cells (Figure 3A–D). This effect was also observed in the Ovc316 cell line after 72 h of drug exposure (Figure 3C). IGROV1-R1 seemed to have a more pronounced effect of *SNHG12* inhibition on carboplatin sensitivity after 72 h than 96 h, although not significant. Exposure to 10 μM carboplatin was not enough to trigger the combined effect with *SNHG12* inhibition on cellular viability. These results indicated that OC cells maintaining a high *SNHG12* expression are more resistant to carboplatin. Therefore, this lncRNA is probably associated with the mechanism of carboplatin resistance in OC cell lines. The effect of siRNA-*SNHG12* was not observed in the IGROV1-R1 cell line at 50 µM of carboplatin exposure (Figure 3B).

## 4. Discussion

A repertoire of molecular and functional studies in OC has been useful for tumor stratification predicting different clinical outcomes [10,52,53,54]. DNA methylation profiling allowed the identification of three molecular subgroups with significant survival differences in HGSCs [55]. DNA methylation of specific genes has shown potential for monitoring disease progression and response to treatment [56]. Methylation of the *BRCA1* promoter is recognized as a somatic driver in approximately 11% of HGSCs, and its loss was associated with acquired chemoresistance [10,52]. In a platinum-sensitive OC cohort mostly comprised of patients with HGSC, the combination of *CAMK2N1* (calcium/calmodulin dependent protein kinase II inhibitor 1) and *RUNX3* (RUNX family transcription factor 3) methylation in tumor specimens was associated with shorter OS and PFS [57]. Additionally, dysregulated DNA methylation may interfere with the expression of lncRNAs in OC, disrupting many processes, including tumor suppressive and oncogenic activities [58,59,60].

In this study, we evaluated the interplay between the DNA methylome and transcriptome profiles in carboplatin-resistant OC cell lines and validated our findings with functional assays and gene expression in OC tissues from HGSC patients. We focused on identifying lncRNA genes both differentially expressed and affected by methylation changes associated with the development of platinum-resistance in OC. An integrative analysis using an external cross-validation set of selected OC samples from TCGA revealed 570 DEGs, including 50 lncRNAs. In this last group, 11 lncRNAs were also associated with at least 1 DMP. Among these 11 lncRNAs, *SNHG12*, *EMX2OS* (EMX2 opposite strand/antisense RNA), and *AC010894.3* had their roles previously investigated in OC, but the mechanisms involved in the transcriptional regulation were barely explored [61,62,63,64]. Our DNA methylation analysis showed that *EMX2OS* exhibited a trend towards hypermethylation in resistant OC cells, while *AC010894.3* was hypermethylated in the same cell lines. *EMX2OS* was previously reported as upregulated in OC tissues and cell lines, enhancing proliferation, invasion, and sphere formation in vitro and tumor growth in vivo [63]. Based on a risk score model, *AC010894.3* was included in a four-lncRNA signature that accurately predicted the OS of OC patients, and its increased expression was associated with better survival [62]. Three other lncRNAs from our gene list, *AC244502.1*, *AP002807.1*, and *AP002518.2*, were integrated into prognostic signatures for breast cancer, clear cell renal cell carcinoma, and Wilms tumor, respectively [65,66,67]. The lncRNA *SNHG12* was selected as a candidate due to its inverse correlation between high expression levels and DNA hypomethylation in carboplatin-resistant OC cell lines.

*SNHG12* is a lincRNA (long intergenic non-coding RNA) and a member of non-coding genes that host small nucleolar RNAs (snoRNA). Approximately 30 *SNHG* genes were identified in the human genome, and some of them (*SNHG1, SNHG3, SNHG5, SNHG6, SNHG7, SNHG12, SNHG15, SNHG16,* and *SNHG20*) have been reported as overexpressed in different types of human cancers [68]. Differently from snoRNAs, which arise exclusively from introns and are classified as small ncRNAs, the transcripts from *SNHG* genes are processed and contain exons. Additionally, *SNHG* RNAs differ from snoRNAs by their subcellular location both in the nucleus and the cytoplasm. Specifically, interactions between *SNHG12* and microRNAs have been implicated in several cancer-related cellular processes and signaling pathways. Experimental analysis showed that *SNHG12* is an endogenous sponge for microRNAs in cervical, prostate, gastric, breast, lung, colorectal cancer, gliomas, osteosarcoma, hepatocellular, papillary thyroid, and clear cell renal cell carcinomas (reviewed in [68,69]). The diversity of interactions with microRNAs showcased the complex molecular network influenced by *SNHG12* and indicated that it might serve as a new therapeutic target in human cancers.

Significantly higher expression levels of *SNHG12* were described in hepatocellular, papillary thyroid, and cervical carcinomas compared to normal tissues, among other cancer types [70,71,72,73]. Association of *SNHG12* expression level with advanced FIGO stage, vascular involvement, and lymph node metastasis in cervical cancers evidenced its potential clinical significance [70]. *SNHG12* overexpression predicted poor prognosis in prostate cancer [74,75], while it was associated with tumor progression and poor survival rates in gastric and lung cancer [76,77]. In our internal dataset, HGSC tissues (*n* = 39) showed higher *SNHG12* expression levels compared to normal ovarian samples (*n* = 5); however, no statistical significance was detected with histopathological and clinical parameters, probably due to the limited number of cases. Sun and Fan evaluated the expression levels of *SNHG12* by RT-qPCR in 24 matched OC and adjacent normal tissues and reported that *SNHG12* levels were higher for patients with stage III-IV than for those with stage I-II, as well as for metastatic patients [61]. Additionally, the 5-year survival analysis was worse in OC patients with high-level relative to those with low-level *SNHG12* expression. Using the OC dataset from TGCA, we found high *SNHG12* expression levels associated with decreased PFS (Figure S3). Altogether, these findings indicate that *SNHG12* is a biomarker of poor prognosis in OC.

We used a siRNA strategy to inhibit *SNHG12* and verified the reduced cell viability of resistant OC cell lines exposed to carboplatin treatment. A previous study detected that A2780 and HO8910 OC cell lines overexpressed *SNHG12* while miR-129 was repressed [61]. The authors demonstrated that *SNHG12* promotes the proliferative and migratory abilities of those OC cells by sponging the miRNA-129 and releasing its target, the *SOX4* (SRY-box transcription factor 4) mRNA. In vitro studies also showed the effect of *SNHG12* repression in inhibiting proliferation, migration and invasion, and metastasis in osteosarcomas and gastric cancer [78,79]. The suppression of *SNHG12* also induced apoptosis and cell cycle arrest, while the opposite effects were observed after the forced expression of *SNHG12* [63]. Similar findings were reported in breast cancer [80]. Of note, *SNHG12* is a downstream target gene of the oncoprotein MYC and its upregulation mediates cell proliferation and migration in triple-negative breast cancer [81].

The mechanism by which *SNHG12* mediates the multi-drug chemoresistance is yet unclear. However, it is recognized that the *SNHG12* knockdown can reverse the resistance to cisplatin, paclitaxel, and gefitinib in cell lines derived from lung cancer [77]. *SNHG12* mediated doxorubicin resistance in osteosarcoma, while its knockdown contributed to the upregulation of miR-320a and improved the sensitivity to this drug [82]. In gliomas, hypomethylation of the promoter region of *SNHG12* was correlated with the transcription factor SP1, leading to its up-regulation in temozolomide-resistant cell lines [83]. In renal cell carcinomas, *SNHG12* was involved in the regulation of the *CDCA3* (cell division cycle associated 3) gene mediated by SP1, promoting resistance to sunitinib [84]. These data indicate that *SNHG12* dysregulation is involved in tumorigenesis, impacts diverse cellular processes, and promotes resistance to chemotherapy.

In addition to the cytoplasm, *SNHG12* was also detected in the nucleus, nucleoplasm, nucleolus, and chromatin [47]. Importantly, regulatory relationships were predicted in a genome region spanning 350Kb that flanks the *SNHG12* locus and includes six genes mapped upstream (*PHACTR4, RCC1, TRNAU1AP*) and downstream (*TAF12*, *RAB42*, and *GMEB1*) [48]. *SNHG12* expression correlates with genes mapped within this region in the 111 OC samples. Our RNA-seq data showed that while the *SNHG12* was upregulated in carboplatin-resistant OC cell lines, the neighboring genes (*PHACTR4*, *RCC1*, *TAF12,* and *GMEB1*) were downregulated in those cells (Figure S4). This finding indicates that *SNHG12* may act as a guide of transcription factors and other protein complexes, such as those involved in cis-regulatory epigenetic mechanisms. This hypothesis is further supported by experimental evidence curated in bioinformatic tools. Co-expression networks demonstrate that *SNHG12* is correlated with genes encoding transcriptional activators such as *SP1* (Sp1 transcription factor), *MYC* (MYC proto-oncogene, bHLH transcription factor), and *STAT2* (signal transducer and activator of transcription 2) in OC and predict interactions between *SNHG12* and HDACI9 protein by Cross-Linking and ImmunoPrecipitation (CLIP) experiments [49,85,86]. The *HDAC9* (histone deacetylase 9) gene is overexpressed in cancer cells and is a member of a family of enzymes responsible for the deacetylation of lysine residues, a key event in the aberrant epigenetic repression in cancer [87]. Other *SNHG* genes, including *DANCR* (former *SNHG13*), *SNHG14*, and *SNHG15,* act synergistically with EZH2 (a histone methylase and catalytic subunit of the Polycomb Repressive Complex 2) to inhibit the expression of downstream target genes in endocrine-related cancers [69]. Recently, Huang et al. surveyed the literature on platinum resistance published in the last 30 years and compiled a list of 937 platinum response-associated genes/proteins. Comparing this set of genes with our RNA-seq and DNA methylation results, we verified that 254 DEGs and 142 DMPs overlapped with entries on the list [51]. The genes encoding *HDAC1* (histone deacetylase 1) and DNA demethylase *TET1* (tet methylcytosine dioxygenase 1) were DEGs, and the *EZH2* (enhancer of zeste 2 polycomb repressive complex 2 subunit) gene associated with DMPs was also DEG in carboplatin-resistant cell lines. Previous studies reported that *EZH2* was generally overexpressed and implicated in the advanced stage, platinum resistance, and poor patient survival in OC [88]. Taken together, these data demonstrate an intrinsic connection between lncRNAs and disrupted epigenetic regulation of gene expression associated with carboplatin-resistance.

## 5. Conclusions

The integrative transcriptomic and epigenomic approach revealed that *SNHG12* is differentially expressed and methylated in OC cells. *SNHG12* modulates carboplatin-response in the cell lines tested, although the mechanism involved in this process should be deeper studied. The plethora of cancer-related features associated with the disrupted expression of *SNHG12* can be a result of the complex network interactions of this lncRNA with miRNAs and proteins. While DNA methylation changes explain the aberrant expression of *SNHG12* in cancer cells, emerging data indicate that *SNHG12* may control the expression of neighboring genes probably by changing epigenetic marks on histones and affecting their transcription.

## Data Availability

The data discussed in this publication have been deposited in NCBI’s Gene Expression Omnibus (GEO) database and are accessible through GEO Series accession number GSE198077 (https://www.ncbi.nlm.nih.gov/geo/query/acc.cgi?acc=GSE198077, accessed on 10 January 2022).

## Supplementary Materials

The following supporting information can be downloaded at: https://www.mdpi.com/article/10.3390/cancers14071664/s1, Figure S1: Workflow of the steps used to evaluate the effects of *SNHG12* knockdown and response to carboplatin in four ovarian cancer cell lines (IGROV1, IGROV1-R1, Ovc316, and OVCAR8); Figure S2: The establishment of carboplatin-resistant cell lines; Figure S3: Kaplan–Meier survival curves of progression-free survival (PFS) and overall survival (OS) according to the expression level of the lncRNA *SNHG12* in ovarian cancer patients; Figure S4: Differential expression of genes in the *SNHG12* locus and encoding components of the epigenetic machinery; Table S1: Mutational status of four cancer driver genes (*TP53*, *BRCA1*, *BRAC2*, and *KRAS*) and carboplatin response of selected cell lines and its carboplatin-resistant counterparts; Table S2: Clinical and pathological features of 39 patients with high-grade serous ovarian carcinoma included in this study; Table S3: Pathway enrichment analysis of differentially expressed genes between carboplatin-sensitive and carboplatin resistant OC derived cells; Table S4: Differentially expressed lncRNAs and overlapping differentially methylated positions in ovarian cancer cell lines relative to its carboplatin-resistant counterparts; Table S5: Differentially expressed genes and differentially methylated positions associated with platinum response.
