# Supplementary material for: The Long Non-Coding RNA SNHG12 as a Mediator of Carboplatin Resistance in Ovarian Cancer via Epigenetic Mechanisms
Source: Cancers (Basel). 2022 Mar 25;14(7):1664. doi: 10.3390/cancers14071664 (PMC8996842; doi:10.3390/cancers14071664)
Supplement: Supplementary file 1 [file cancers-14-01664-s001.zip › Supplementary material_Figures S1, S2, S3. and S4.pdf]

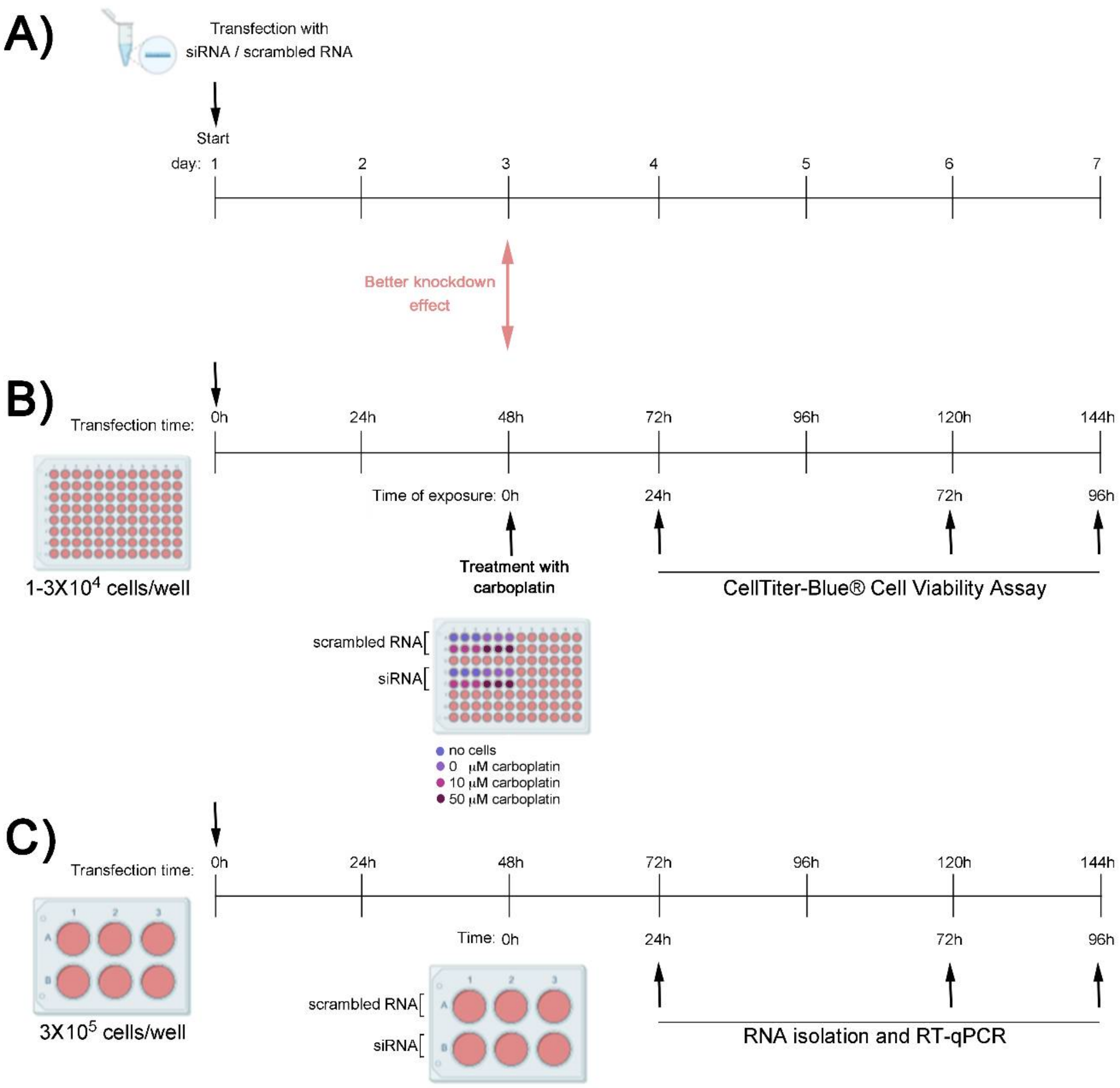

Figure S1. Workflow of the steps used to evaluate the effects of *SNHG12* knockdown and response to carboplatin in four ovarian cancer cell lines (IGROV1, IGROV1-R1, Ovc316, and OVCAR8). **A**) Transfection with *SNHG12* siRNA or scrambled RNA was performed in triplicate and two independent experiments. **B**) Cell viability was measured using CellTiter Blue Cell Viability assay (Promega, Madison, USA) after 24h, 72h, and 96h of carboplatin treatment at two concentrations (10μM and 50μM). **C**) Confirmation of the transient knockdown was monitored at the same time points by the expression analysis by RT-qPCR.

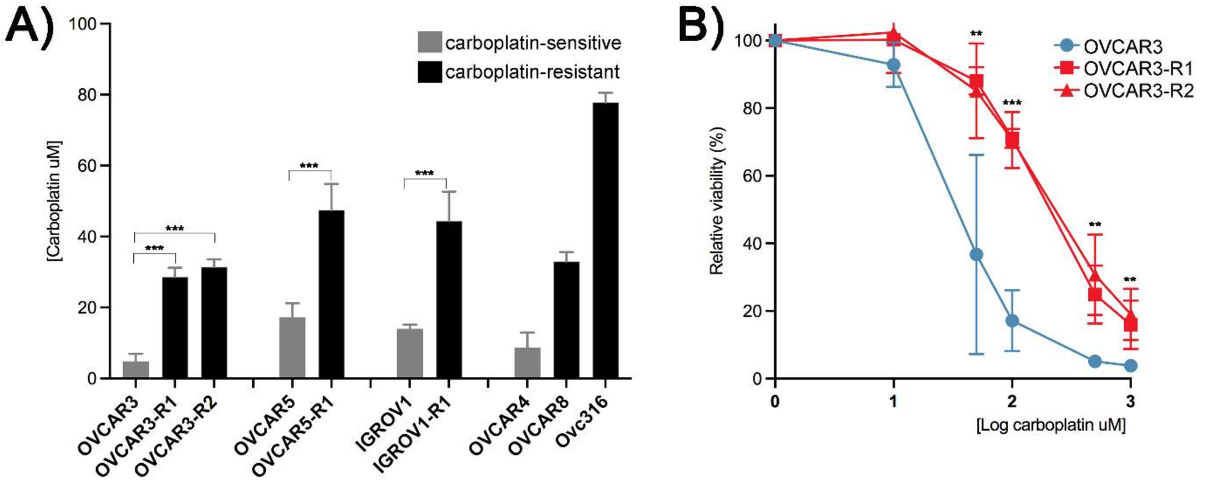

Figure S2. The establishment of carboplatin-resistant cell lines**. A)** IC_{50} values of the ovarian cancer cell lines included in this study. The resistant cell lines derived from OVCAR3, OVCAR5, and IGROV1 are designated R1 or R2. The IC_{50} was quantified using a crystal violet assay following six days of treatment with carboplatin (1-100µM). The measurements were performed in duplicates. The IC_{50} values represent the mean of 3-5 independent viability experiments ± standard deviation (* $p < 0.05$; ** $p < 0.01$; *** $p < 0.001$). **B)** Carboplatin dose-response curves of OVCAR3 and its carboplatin-resistant derivatives R1 and R2.

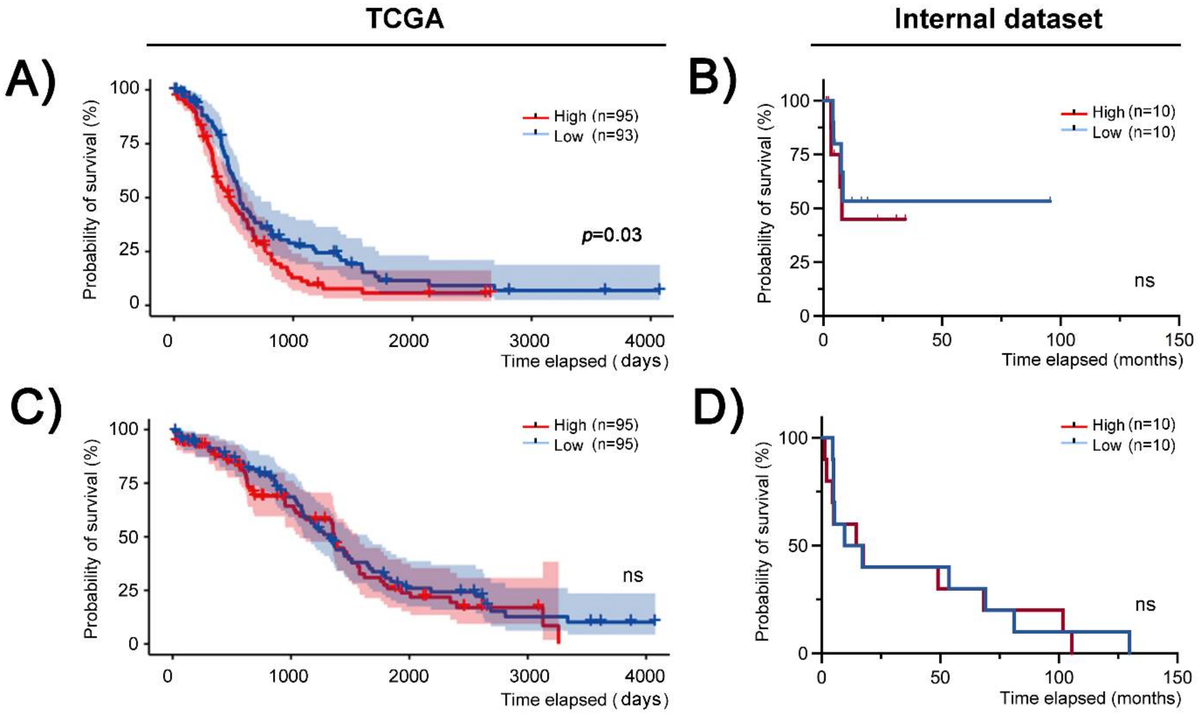

Figure S3. Kaplan–Meier survival curves of progression-free survival (PFS) and overall survival (OS) according to the expression level of the lncRNA *SNHG12* in ovarian cancer patients retrieved from TCGA available on Lnc2Cancer 3.0 database (http://bio-bigdata.hrbmu.edu.cn/lnc2cancer/); **A** and **C**, respectively) and the internal cohort used in this study (**B** and **D**, respectively). ns = not significant.

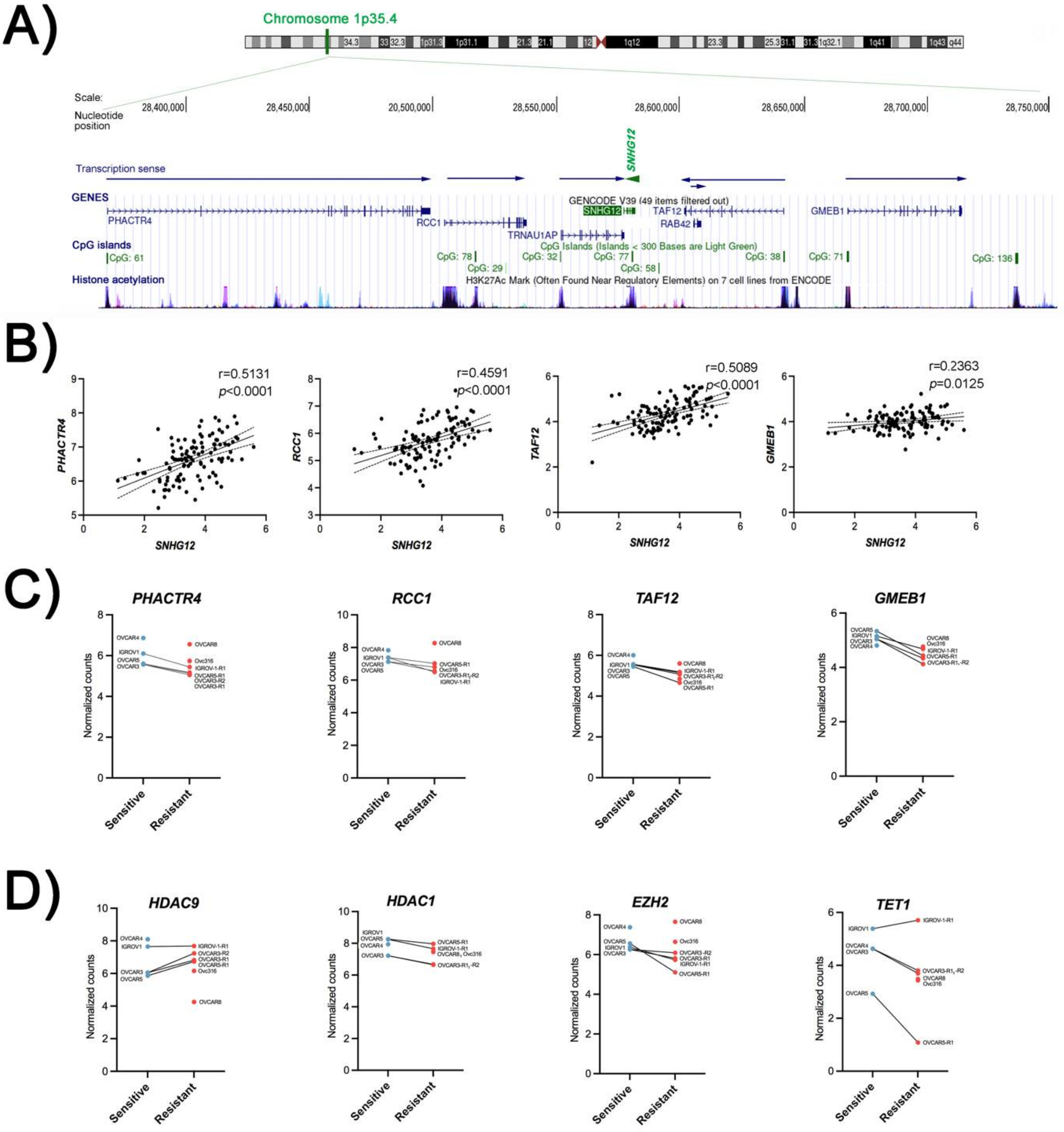

Figure S4. Differential expression of genes in the *SNHG12* locus and encoding components of the epigenetic machinery. **A**) Genomic architecture of the *SNHG12* locus. Scheme of the genes, CpG islands, and acetylation levels of lysine 27 of histone H3 organized according to their physical location on 1p35.4 (UCSC Genome Browser on Human, assembly ID: hg38, http://genome.ucsc.edu). **B)** Pearson correlation demonstrating putative regulatory relationships between genes expression levels mapped within a 350kb region flanking lncRNA *SHG12* in 111 ovarian cancer samples from TCGA. **C**) Differential gene expression among carboplatin-sensitive and carboplatin-resistant ovarian cancer cell lines. The genes *PHACTR4*, *RCC1*, *TAF1*, and *GMEB1* are downregulated in carboplatin-resistant counterparts. The quantification analysis was performed using RNA-sequencing data. Normalized counts are expressed as log2-counts-per-million. **D**) Genes encoding histone modifier enzymes (*HDAC9*, *HDAC1*, and *EZH2*) and DNA demethylase (*TET1*) were also detected as differentially expressed in the same cell lines.
