# Supplementary material for: The Long Non-Coding RNA SNHG12 as a Mediator of Carboplatin Resistance in Ovarian Cancer via Epigenetic Mechanisms
Source: Cancers (Basel). 2022 Mar 25;14(7):1664. doi: 10.3390/cancers14071664 (PMC8996842; doi:10.3390/cancers14071664)
Supplement: Supplementary file 1 [file cancers-14-01664-s001.zip › Supplementary material_Tables.pdf]

Table S1. Mutational status of four cancer driver genes (*TP53*, *BRCA1*, *BRAC2*, and *KRAS*) and carboplatin response of selected cell lines and its carboplatin-resistant counterparts.

Cell line	Mutation status			Carboplatin IC_{50} (µM)
	TP53	***BRCA1/2***	***KRAS***	
IGROV1	p.Y126C	*BRCA1* p.K654fs*47<br>*BRCA2* p.P3150T	wt	14.0 ± 1.1
IGROV1-R1	p.Y126C	*BRCA1* p.K654fs*47<br>*BRCA2*_p.P3150T	wt	44.4 ± 8.2
OVCAR3	p.R248Q	wt	wt	4.8 ± 2.1
OVCAR3-R1	p.R248Q	wt	wt	28.6 ± 2.6
OVCAR3-R2	p.R248Q	wt	wt	31.4 ± 2.1
OVCAR4	p.L130V	*BRCA2*_p.P2505L	wt	8.8 ± 4.2
OVCAR5	wt	wt	p.Gly12Val	17.2 ± 4.0
OVCAR5-R1	wt	wt	p.Gly12Val	47.4 ± 7.4
OVCAR8	c.376-1G>A<br>(splice variant)	wt	wt	33.0 ± 2.6
Ovc316	-	wt	wt	77.8 ± 2.8

R1 and R2 carboplatin-resistant derived counterparts;
wt: wide type, no predicted deleterious mutation identified.

Table S2. Clinical and pathological features of 39 patients with high-grade serous ovarian carcinoma included in this study.

**Variable**	**Cases (N=39)**
**Age at diagnosis** (years)	
Mean (±Std. Deviation)	69.4± 9.4
Median	69.1
Range	min 46 - max 83
**FIGO stage**	
III	31
IV	8
**CA-125 baseline level, U/L***	
Median	803
Range	min 179 - max 17096
**Progression-free survival** (months)	
0-6	11
7-11	9
>12	19
**Overall survival** (months)	
<12	12
>12	27

(*) data available on 22 out of 39 cases.

Table S3. Pathway enrichment analysis of differentially expressed genes between carboplatin-sensitive and carboplatin resistant OC derived cells.

Pathway name	Entities				Reactions	
	**found**	**ratio**	***p*-value**	**FDR***	**found**	**ratio**
**Up-regulated genes**						
Glutathione conjugation	4/67	0.006	0.003	0.268	1/14	0.001
Activation of PUMA and translocation to mitochondria	2/10	6.97e-04	0.003	0.268	5/5	3.78e-04
Formyl peptide receptors bind formyl peptides and many other ligands	2/11	7.47e-04	0.004	0.268	2/3	2.27e-04
Signaling by TGFB family members	4/114	0.008	0.016	0.47	11/107	0.008
FOXO-mediated transcription of cell death genes	2/23	0/002	0.016	0.47	2/15	0.001
TP53 regulates transcription of genes involved in cytochrome C release	2/33	0.002	0.032	0.47	2/25	0.002
Antagonism of activin by follistatin	1/4	2.72e-04	0.033	0.47	2/2	1.51e-04
TGFBR1 LED mutants in cancer	1/4	2.72e-04	0.033	0.47	1/1	7.56e-05
Signaling by TGF-beta receptor complex	3/85	0.006	0.035	0.47	4/82	0.006
Activation of BH3 only proteins	2/36	0.002	0.037	0.47	5/19	0.001
SMAD2/SMAD3:SMAD4 heterotrimer regulates transcription	2/39	0.003	0.043	0.47	1/17	0.001
BMAL1:CLOCK, NPAS2 activates circadian gene expression	2/42	0.003	0.049	0.47	2/20	0.002
**Down-regulated genes**						
RUNX1 regulates genes involved in megakaryocyte differentiation and platelets function	11/73	0.005	1.13e-07	6.70e-05	21/33	0.002
RNA Polymerase I promoter opening	8/34	0.002	1.43e-07	6.70e-05	2/2	1.51e-04
IGF1R signaling cascade	10/72	0.005	4.92e-07	1.30e-04	9/17	0.001
Activated PEN1 stimulates transcription of AR (androgen receptor) regulated genes KLK2 and KLK3	8/49	0.003	2.15e-06	4.02e-04	9/11	8.32e-04
**DNA methylation**	**7/36**	**0.002**	**3.04e-06**	**4.74e-04**	**7/7**	**5.29e-04**
NoRC negatively regulates rRNA expression	9/80	0.005	9.89e-05	0.001	5/7	5.29e-04
**PRC2 methylates histones and DNA**	**7/44**	**0.003**	**1.11e-05**	**0.001**	**4/4**	**3.02e-04**
SIRT1 negatively regulates rRNA expression	7/45	0.003	1.28e-05	0.001	3/5	3.78e-04
Unfolded protein response (UPR)	12/15 5	0.011	1.44e-05	0.001	13/94	0.007
RNA Polymerase I promoter escape	8/64	0.004	1.47e-05	0.001	2/2	1.51e-04
Signaling by FGFR	10/10 7	0.007	1.56e-05	0.001	777/142	0.011
ERCC2 (CSB) and EHMT2 (G9a) positively regulate rRNA expression	7/48	0.003	1.92e-05	0.001	2/4	3.02e-04

Table S4. Differentially expressed lncRNAs and overlapping differentially methylated positions in ovarian cancer cell lines relative to its carboplatin-resistant counterparts.

lncRNA	Chr	Nucleotide position		Gene expression analysis		DNA methylation analysis		
		Start	End	log FC	*p*-value	Probe ID	Δβ	*p*-value
SNHG12	1	28578538	28583132	0.41	0.010	cg08625918	-0.08	0.03
LINC01781	1	80535755	80646791	-1.65	0.003	cg22327746	0.24	0.03
AC010894.3	2	174547141	174776720	-1.15	0.047	cg17095939	0.16	0.02
						cg14797460	0.04	0.00
						cg08213000	0.17	0.01
						cg15174367	0.05	0.03
LINC01811	3	33956972	34677247	1.48	0.004	cg17661563	0.36	0.05
AC025470.2	5	57395060	57534504	1.49	0.002	cg00325378	0.24	0.04
LINC01277	6	142966293	143059682	0.75	0.009	cg19979228	0.22	0.03
						cg23117546	0.35	0.02
						cg03616157	0.20	0.01
EMX2OS	10	117473215	117545068	-0.54	0.034	cg04556008	0.39	0.03
AP002807.1	11	68050740	68053762	0.90	0.001	cg00387390	0.12	0.04
						cg14774134	0.07	0.00
						cg15085109	0.08	0.02
AP002518.2	11	114210616	114356571	0.81	0.047	cg03409036	0.11	0.04
AC244502.1	14	22197446	22482959	1.09	0.009	cg21875948	0.37	0.03
						cg10268229	0.21	0.01
AC087612.1	15	63091085	63110589	4.06	8.93e-12	cg06828657	0.17	0.02

Chr: chromosome; logFC: log Fold-Change
